# Multi-Modal Data Fusion for 3D Object Detection Using Dual-Attention Mechanism

**DOI:** 10.3390/s25206360

**Published:** 2025-10-14

**Authors:** Mengying Han, Benlan Shen, Jiuhong Ruan

**Affiliations:** 1 School of Rail Transportation, Shandong Jiaotong University, Jinan 250357, China; hanmengying@stu.sdjtu.edu.cn (M.H.); ruanjh2011@163.com (J.R.); 2 Key Laboratory of Rail Transit Safety Technology and Equipment, Shandong Province Transportation Industry, Jinan 250357, China

**Keywords:** 3D object detection, multimodal data fusion, pillar-wise channel attention, spatial attention mechanism

## Abstract

To address the issue of missing feature information for small objects caused by the sparsity and irregularity of point clouds, as well as the poor detection performance on small objects due to their weak feature representation, this paper proposes a multi-modal 3D object detection method based on an improved PointPillars framework. First, LiDAR point clouds are fused with camera images at the data level, incorporating 2D semantic information to enhance small-object feature representation. Second, a Pillar-wise Channel Attention (PCA) module is introduced to emphasize critical features before converting pillar features into pseudo-image representations. Additionally, a Spatial Attention Module (SAM) is embedded into the backbone network to enhance spatial feature representation. Experiments on the KITTI dataset show that, compared with the baseline PointPillars, the proposed method significantly improves small-object detection performance. Specifically, under the bird’s-eye view (BEV) evaluation metrics, the Average Precision (AP) for pedestrians and cyclists increases by 7.06% and 3.08%, respectively; under the 3D evaluation metrics, these improvements are 4.36% and 2.58%. Compared with existing methods, the improved model also achieves relatively higher accuracy in detecting small objects. Visualization results further demonstrate the enhanced detection capability of the proposed method for small objects with different difficulty levels. Overall, the proposed approach effectively improves 3D object detection performance, particularly for small objects, in complex autonomous driving scenarios.

## 1. Introduction

In recent years, autonomous driving technology has undergone rapid advancements. The current research on autonomous driving is a multidisciplinary field that integrates various domains, including motor power [1], intelligent sensing, image processing, and intelligent control. As a fundamental component of autonomous driving systems, environmental perception plays a pivotal role in enabling information exchange between vehicles and the surrounding environment. Its primary objective is to equip autonomous vehicles with perception capabilities comparable to or even exceeding those of human drivers, allowing them to accurately perceive and interpret the dynamic driving environment. To achieve accurate perception, modern autonomous driving systems typically employ multiple onboard sensors, including cameras, LiDAR, and millimeter-wave radar [2]. Cameras provide low-cost sensing and semantically rich information that closely resembles human visual perception. However, their performance is highly susceptible to lighting and environmental conditions, and they inherently lack depth information. LiDAR provides accurate and high-resolution 3D spatial information through point clouds, but it cannot capture texture and color details [3]. Millimeter-wave radar offers reliable distance measurement under adverse weather conditions such as rain, fog, and dust, making it suitable for all-weather perception. However, the resulting point clouds are sparse, limiting their capability to precisely characterize object geometries [4]. With the rapid progress of perception technology, 3D object detection has become a central research focus in computer vision and autonomous driving. Compared with traditional 2D object detection, 3D object detection not only identifies object categories but also estimates their 3D position, size, and orientation with high accuracy, thereby offering richer environmental context for downstream tasks such as path planning and motion prediction. Among various 3D perception modalities, LiDAR-generated point clouds are widely adopted in 3D object detection due to their rich spatial information and robustness to lighting variations.

According to the manner in which point cloud data is processed, existing 3D object detection approaches can be broadly classified into three categories: point-based, voxel-based, and multi-view-based methods. Point-based [5] methods directly extract features from raw point cloud data, thereby preserving geometric structures as much as possible. Representative methods, such as PointNet++ [6], introduce hierarchical structures for extracting local region features, thereby improving geometric detail representation. PointRCNN [7] follows a two-stage framework that directly generates candidate bounding boxes from the raw point cloud and refines them to achieve high detection accuracy. Pointformer [8] leverages the Transformer module to model long-range dependencies, thereby enhancing its capability for global modeling of sparse point clouds. These methods perform well in detecting small objects but are often limited by high computational complexity and low inference efficiency.

Voxel-based methods partition point clouds into regular 3D voxel grids, followed by 3D convolutions for feature extraction. VoxelNet [9] was the first to propose encoding point cloud data within each voxel using PointNet [10], combined with 3D convolutions for end-to-end training. SECOND [11] further introduced sparse convolution to reduce computational redundancy and significantly improve inference speed. PointPillars [12] generates BEV representations by partitioning point clouds into vertical columns, which are processed using lightweight 2D convolutional networks to substantially improve detection efficiency. This method has been widely adopted in real-world engineering applications. PV-RCNN [13] enhances the detection of small objects by jointly exploiting point- and voxel-level features. PV-RCNN++ [14] extends this framework by refining point–voxel feature fusion through local vector representations. PillarNeXt [15] advances detection performance in complex scenarios with improved pillar encoding and multi-scale feature aggregation. Voxel-based methods offer regular data structures and strong parallelism, making them well-suited for large-scale scene processing; however, they face performance bottlenecks in capturing fine edge details and detecting small objects.

Multi-view methods project point clouds onto multiple 2D views (e.g., BEV, front view, and depth maps), followed by 2D convolutional detection networks. Representative approaches, such as MV3D [16], fuse multi-view information to enhance detection performance. In recent years, multi-view detection methods have progressively advanced toward more efficient BEV representations and temporal modeling. BEVDet [17] projects features from multiple camera views into a unified BEV space, enabling efficient 3D detection. Building upon this, BEVDet4D [18] incorporates temporal information to improve robustness in dynamic scenes. Meanwhile, PETRv2 [19] leverages the Transformer architecture with positional encoding to achieve end-to-end BEV representation learning, further enhancing the spatial features of multi-view images. These methods leverage mature 2D detection architectures, achieving high detection efficiency and accuracy in specific scenarios. However, the dimensionality reduction process can lead to the loss of semantic and geometric information, especially when representing complex spatial structures. In summary, although each method offers unique advantages in structural design and performance optimization, they all face common challenges, including poor representation of small-object features caused by point cloud sparsity and inadequate fusion of geometric and semantic information.

To further address the limitations of the aforementioned methods, recent studies have extensively explored fusing camera images with LiDAR point clouds to improve 3D object detection performance. Depending on the fusion stage, existing multimodal fusion strategies are generally categorized into three types: early fusion, mid-fusion, and late fusion. Early fusion projects image features onto point clouds via geometric relationships, thereby achieving data-level information complementarity. In contrast, mid-fusion methods (e.g., BEVFusion [20]) integrate information through feature-level interactions following modality-specific encoding. While these approaches often deliver strong overall performance, they face challenges in cross-modal alignment and computational efficiency, and the semantic features of small objects may be weakened during convolution and feature aggregation. Late fusion methods (e.g., TransFusion [21]) perform decision-level integration at the stage of detection results or high-level semantics, providing robustness but limited enhancement of low-level features, thereby reducing their ability to preserve small-object semantics. Motivated by these observations, this paper adopts the PointPainting [22] data-level fusion strategy, which effectively enhances the semantic representation of small objects at the early feature level and provides more comprehensive input information for subsequent detection networks.

Beyond the fusion of camera images and LiDAR point clouds, recent advances in vision-based object detection and neural network design have provided valuable insights for small-object detection. For example, Zhang et al. [23] proposed a lightweight and efficient convolutional neural network for industrial surface defect detection, demonstrating that lightweight CNNs can achieve high-speed inference while maintaining competitive accuracy. Qin et al. [24] introduced an unsupervised image stitching approach based on generative adversarial networks and a feature frequency awareness algorithm, improving the quality and robustness of image features through multi-scale feature collaboration and contextual information fusion, thus providing useful guidance for multimodal feature alignment. Moreover, Zhang et al. [25] developed a lightweight network for underwater object detection that effectively enhances small-object detection via an attention-guided cross-scale feature interaction module, achieving a balance between efficiency and robustness in complex underwater environments. Collectively, these studies offer critical insights for improving small-object detection and facilitating adaptation to complex environments in autonomous driving scenarios.

Although data-level semantic fusion has demonstrated notable improvements in enhancing the semantic representation of point clouds, small objects remain vulnerable to information loss during feature extraction and propagation. This is primarily due to the inherent sparsity of their corresponding point clouds and limited feature representation capacity. In particular, when the fused point clouds are encoded into a pseudo-image feature representation, essential features may be further degraded as a result of projection compression, ultimately degrading detection performance. To address these issues, this paper proposes an enhanced PointPillars framework tailored for small-object detection in autonomous driving scenarios, aiming to improve overall detection performance. The primary contributions of this work are as follows:

(1) The PointPainting-based data-level fusion strategy is adopted to fuse image semantic information into the original point clouds, thereby generating semantically enriched point clouds to improve the semantic representation of small objects.
(2) Before generating pseudo-image representations within the pillar feature encoding network, the PCA module is incorporated to improve the feature representation of small objects. This module adaptively learns the importance of channels and features, assigning higher weights to those that contribute more significantly to the detection task while reducing the influence of less relevant information.
(3) To address the limitations of feature extraction capability, the SAM is integrated into the backbone network to emphasize critical regions while suppressing background noise via spatial attention weights.
(4) Quantitative and qualitative evaluations conducted on the KITTI dataset demonstrate that the proposed algorithm significantly outperforms existing methods in detecting small objects, thereby enhancing object detection performance.

In summary, the algorithm proposed in this paper effectively enhances the performance of 3D object detection for small objects in autonomous driving scenarios by employing a data-level fusion strategy that integrates camera images with LiDAR point clouds and by introducing channel and spatial attention mechanisms. These improvements not only advance the development of 3D object detection technology but also provide a more reliable perceptual foundation for downstream modules such as path planning and motion prediction in autonomous driving systems.

## 2. Materials and Methods

**Figure 1:**
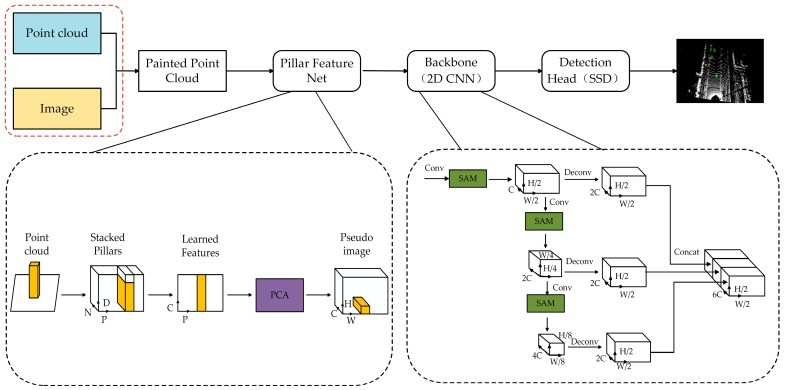
Overall architecture of the improved algorithm; PCA: Pillar-wise Channel Attention module; SAM: Spatial Attention Module.

The overall architecture of the improved model proposed in this paper is illustrated in Figure 1. It employs PointPainting’s data-level multimodal fusion strategy. Semantic information is extracted from images via a pre-trained semantic segmentation network and projected onto the point cloud using camera–LiDAR calibration parameters [22], thereby assigning semantic labels to each point and generating painted point clouds as input for detection. Secondly, to further enhance feature representation, the PCA module is introduced prior to the generation of pseudo-image representations within the PointPillars’ PFN layer. This module adaptively learns the relative importance of features across different channels, effectively enhancing the network’s ability to focus on key features and mitigating the feature loss of small objects after conversion to pseudo-image representations. Finally, SAM is integrated into the backbone network to enhance its focus on target regions while suppressing redundant background interference, thereby improving feature extraction for small objects and boosting the model’s perception and detection performance in complex scenarios.

### 2.1. Image and Point Cloud Data Fusion Strategy

**Figure 2:**
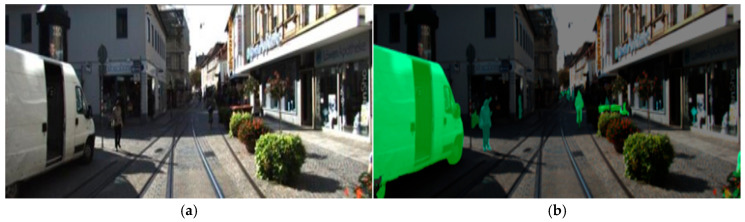

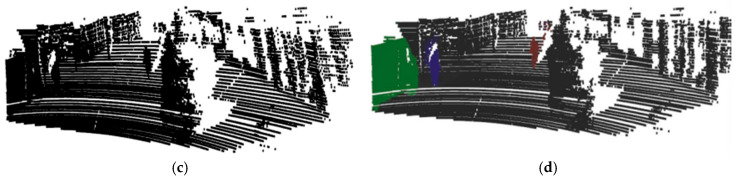
These are the effect pictures before and after the point cloud fusion: (**a**) Original picture; (**b**) Semantic segmentation result; (**c**) Original point cloud; (**d**) Fused point cloud.

LiDAR point clouds generally possess higher density and resolution than the point clouds generated by millimeter-wave radar. However, the point cloud data corresponding to small objects—such as pedestrians and bicycles—are relatively sparse, which constrains the accuracy of 3D object detection. To address this issue, this paper adopts the data-level fusion strategy of PointPainting, leveraging both LiDAR point clouds and camera images as multimodal inputs for object detection. The proposed algorithm comprises three main stages: (1) Semantic Segmentation: The segmentation scores for each pixel are computed using the DeepLabV3+ network, employing weights pre-trained on the Cityscapes dataset. (2) Fusion: The segmentation scores are projected onto the corresponding LiDAR point cloud, effectively painting the raw point cloud with semantic information. (3) 3D Object Detection: The improved PointPillars network is then utilized to detect objects from the semantically enriched point clouds. By projecting point clouds onto the image plane, PointPainting extracts semantic features from images and maps them back onto the 3D space, achieving data-level fusion of semantic and spatial information. The resulting semantically enriched point clouds improve the network’s perception of small objects. An example of this fusion process on the KITTI dataset is shown in Figure 2.

By incorporating semantic information from images, data-level fusion between camera images and LiDAR point clouds is achieved, producing semantically enriched point clouds that significantly enhances the model’s perception of small objects. This approach fully leverages image semantic and point cloud spatial location information, establishing a robust foundation for subsequent 3D object detection.

### 2.2. Improvements to the Pillar Feature Net

The PointPillars’ Pillar Feature Network (PFN) layer first partitions point clouds into a set of regular pillar units with fixed intervals on the X-Y plane of the Cartesian coordinate system. Each pillar corresponds to a specific spatial location and contains multiple points within its coverage area. Subsequently, points within each pillar are aggregated and fed into a feature extraction network for encoding, and then transformed into a 2D pseudo-image representation via feature mapping, enabling the network to efficiently learn spatial features through a 2D convolutional architecture. However, point clouds are inherently sparse, particularly in distant or occluded regions. Small objects, such as pedestrians and cyclists, often consist of only a very small number of points in the point cloud. Consequently, when the PointPillars’ PFN layer converts the point cloud into pseudo-images, the feature representation of small object regions becomes insufficient, hindering the effective extraction of features from small objects in subsequent convolution operations.

**Figure 3:**
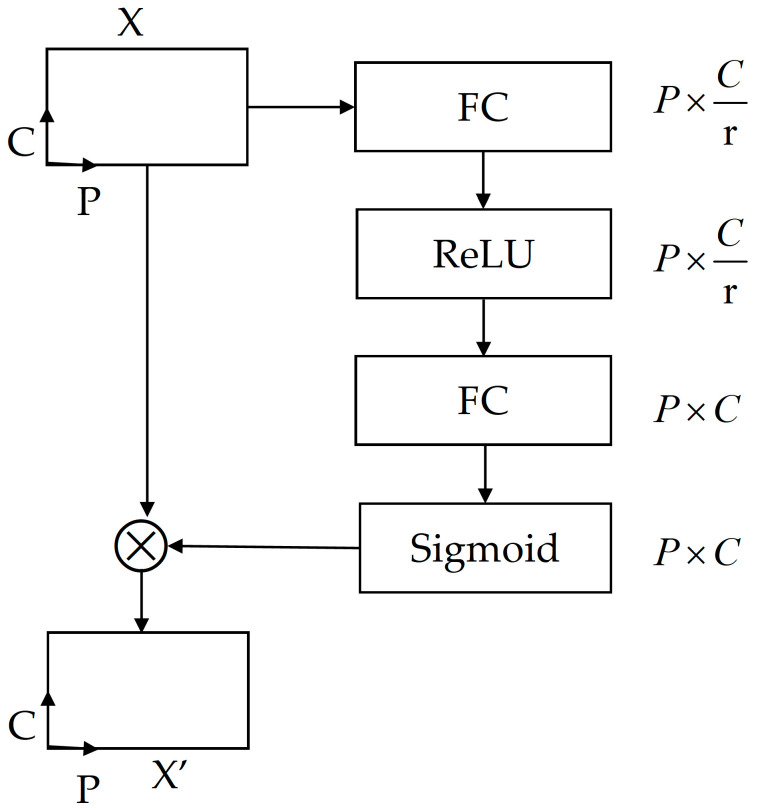
Pillar-wise channel attention.

As shown in Figure 1, the PCA module is introduced before converting the pseudo-image representation in the PFN layer. This module aims to adaptively assign weights to the feature channels within each pillar, thereby enhancing the expressiveness of key channels and suppressing information of redundant channels. Compared to the traditional SE module, the PCA designed in this paper is specifically tailored for sparse point cloud pillar structures. By combining local geometric features within the pillar and channel distribution differences, it more efficiently enhances the channel representation capability of small objects, significantly improving small-object detection performance. The PCA is shown in Figure 3. The input to the module is a pillar feature tensor with a shape of P×C. *P* denotes the number of pillars and *C* represents the number of feature channels. The module first applies a shared fully connected (FC) layer to reduce the channel dimension to C/r. The FC layer is followed by a ReLU activation function to introduce non-linear representation. Then the next FC layer is used to restore the original channel dimension. Subsequently, a Sigmoid activation function is applied to generate channel attention weights, as shown in Equation (1). The output retains the shape of P×C, indicating the importane scores assigned to each channel of each pillar.

(1) $$s=\sigma (W_{2}\delta (W_{1}X))$$

In Formula (1): σ is the Sigmoid function; $W_{1}$, $W_{2}$ is two FC layers weights; δ is the ReLU activation function. Finally, the attention weights are multiplied element-wise with the original features, as shown in Equation (2), to enhance significant features and suppress redundant features, thereby achieving adaptive feature learning.

(2) $$X^{\prime }=s\cdot X$$

The PointPillars’ PFN layer leads to partial loss of point cloud information, resulting in insufficient feature representation in the generated pseudo-image representations. To address this, the PCA module is introduced to enhance focus on critical point cloud features while suppressing irrelevant information, thereby improving feature extraction and boosting 3D object detection accuracy. Additionally, the module features a simple architecture and incurs low computational overhead, satisfying the real-time requirements of autonomous driving systems. It is spatially independent and can be flexibly integrated into existing pillar-based 3D detection frameworks.

### 2.3. Improvements to the Backbone Network

The original PointPillars employs a two-dimensional convolutional neural network to perform multiple downsampling operations on pseudo-image representations, progressively reducing spatial resolution while extracting high-level semantic features. Subsequently, feature representations obtained from downsampling at various scales are upscaled to a uniform size and concatenated to construct multi-scale fused feature representations for object detection. However, when processing small objects, such as pedestrians and cyclists, the network faces issues such as feature weakening and insufficient feature representation.

**Figure 4:**
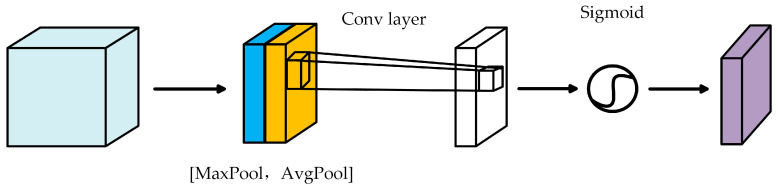
Spatial attention module.

To address these issues, as illustrated in Figure 1, this paper introduces SAM into the backbone network to enhance the attention of key regions in BEV feature representations. The structure of the SAM is depicted in Figure 4. Specifically, this module applies average pooling and max pooling along the channel dimension to generate two spatial feature maps.

(3) $$M_{avg}=AvgPool(X)$$

(4) $$M_{\max}=MaxPool(X)$$

After concatenating the two feature maps, the attention weights in SAM are calculated through a 7 × 7 convolution followed by a sigmoid activation. The 7 × 7 convolution enlarges the receptive field, thereby strengthening the spatial correlation of small targets in sparse feature maps and alleviating the dispersion of attention caused by insufficient local context. The final formulation of this process is given in Equation (5).

(5) $$\begin{array}{l} M_{s}(X)=\sigma \{f^{7\times 7}[AvgPool(X);MaxPool(X)]\}\\ =\sigma [f^{7\times 7}(M_{avg};M_{\max})] \end{array}$$

In the 3D object detection task, incorporating SAM effectively enhances the backbone network’s ability to focus on important spatial regions, thereby improving both detection accuracy and robustness.

## 3. Experimental Setup

### 3.1. Dataset

The method proposed in this paper is evaluated on the publicly available KITTI dataset, which provides images and point clouds covering scenarios such as urban streets, rural roads, and highways. Each image contains at most 15 cars and 30 pedestrians. The dataset consists of 7481 training samples and 7518 test samples. Following the commonly adopted PointPillars data split, the training set is further divided into 3712 training samples and 3769 validation samples. The training set is used to train the model, while the validation set is employed for performance evaluation. Each object category is further divided into three difficulty levels—easy, moderate, and hard—based on object size and degree of occlusion, as shown in Table 1.

### 3.2. Experimental Environment

The experiments were conducted using the OpenPCDet open-source codebase, where the improved PointPillars algorithm was implemented and evaluated on the KITTI dataset. The hardware environment consisted of an NVIDIA RTX A5000 GPU (24 GB) (Santa Clara, CA, USA) and an Intel^®^ Xeon^®^ w5-2445 CPU (Santa Clara, CA, USA). The software environment was composed of Ubuntu 22.04 LTS, Python 3.9, CUDA 11.3, and PyTorch 1.10. The network architecture was implemented in PyTorch, and both training and testing were conducted on the GPU.

### 3.3. Experimental Details

The point cloud voxelization range was defined as [0, −39.68, −3, 69.12, 39.68, 1], with a voxel size of (0.16, 0.16, 4). Each voxel contained up to 32 points. During training, the model processed a maximum of 16,000 voxels per batch, whereas the upper limit during testing was set to 40,000 voxels. Optimization was performed using the Adam OneCycle optimizer with an initial learning rate of 0.003. The model was trained for 120 epochs, with a batch size of 8. The learning rate increased linearly during the first 40% of iterations and then decayed linearly over the remaining 60%. Regarding the loss function, the weights were assigned as follows: the classification loss was weighted by 1.0, the bounding box regression loss by 2.0, the orientation prediction loss by 0.2, and all encoding weights were uniformly set to 1.0. To prevent gradient explosion, gradient clipping with a threshold of 10 was applied during training. In the post-processing stage, non-maximum suppression was performed with a threshold of 0.01, retaining a maximum of 500 candidate bounding boxes. Model evaluation on the validation set was conducted using the AP40 metric, where the IoU threshold for the Car class was 0.7, and the IoU thresholds for the Pedestrian and Cyclist classes were 0.5.

## 4. Results

### 4.1. Quantitative Analysis

The proposed algorithm is evaluated on the KITTI dataset using AP as the primary performance metric. According to the KITTI evaluation protocol, evaluation is conducted based on 40 equally spaced recall positions. For category-wise evaluation, an Intersection over Union (IoU) threshold of 0.7 is applied for cars, while a threshold of 0.5 is used for both pedestrians and cyclists. Furthermore, performance is assessed across multiple metrics, including BEV for bird’s-eye view detection and 3D for three-dimensional bounding boxes.

To evaluate the performance of the proposed algorithm, it is compared with several representative 3D object detection algorithms on the KITTI test set. These algorithms include SECOND, F-PointNet [26], MVX-Net [27], PointPillars, PillarNet [28], SMS-Net [29], and VoxelNextFusion [30]. Table 1 and Table 2 present the average precision of the proposed algorithm and the compared algorithms for the three object categories—Car, Cyclist, and Pedestrian—under both BEV and 3D detection perspectives.

As shown in Table 2 and Table 3, the improved algorithm proposed in this paper demonstrates significant accuracy improvements over the original PointPillars method for detecting cars, pedestrians, and cyclists on the KITTI dataset. Since this model places greater emphasis on feature extraction and the detection of small-sized objects such as cyclists and pedestrians, the accuracy gains for larger objects, such as cars, are slightly smaller. In the BEV detection task, the accuracy for cyclist detection in the moderate difficulty level increases from 66.21% to 69.98%, representing a 3.77% improvement. For pedestrians, the accuracy is improved by 7.92%, 6.74%, and 6.51% across the easy, moderate, and hard difficulty levels, respectively. In the 3D detection task, cyclist detection accuracy increased by 1.94%, 2.85%, and 2.92% at the easy, moderate, and hard levels, respectively, while pedestrian detection accuracy is improved by 5.26%, 4.31%, and 3.52% at the corresponding difficulty levels.

Furthermore, when detecting cars, pedestrians, and cyclists, the proposed algorithm exhibits distinct advantages over other methods. This improvement is achieved by applying a data-level multimodal fusion strategy based on PointPainting within the PointPillars framework, which integrates semantic information from images into the original point clouds to generate painted point clouds. This fusion effectively enriches the feature representation of key objects, thereby enhancing detection accuracy. Additionally, the PCA module is incorporated into the PFN layer of PointPillars to adaptively assign channel weights within each pillar, resulting in richer feature representations for small and medium-sized objects and improving the detection performance of small objects. Finally, SAM is integrated into the backbone network of PointPilars to emphasis critical objects regions, substantially boosting the backbone’s feature extraction capabilities.

### 4.2. Performance Analysis

To enhance the representation capability of pillar-based point cloud features, we introduce a channel attention mechanism that adaptively recalibrates feature responses across channel dimensions. The proposed PCA module is specifically designed with the pillar structure of PointPillars in mind, operating directly on each pillar’s channel dimension to strengthen feature representation. In our experiments, the channel compression ratio r of the PCA module is set to 8, balancing computational efficiency and representational power. In contrast, the conventional SE module applies attention to two-dimensional pseudo-image representations generated from pillar projections. However, due to the sparse distribution and limited number of points associated with small objects, local features can become weakened during the projection from 3D point clouds to 2D pseudo-image representations, which reduces the ability of SE to effectively capture small-object information. As shown in Table 4, when integrating semantic information, the PCA module achieves +1.88% BEV mAP and +2.78% 3D mAP improvements over the SE module for small-object categories (pedestrians and cyclists). These results demonstrate that the PCA module provides a more favorable balance between efficiency and detection performance, while leveraging semantic information more effectively to enhance overall accuracy.

### 4.3. Qualitative Analysis

**Figure 5:**
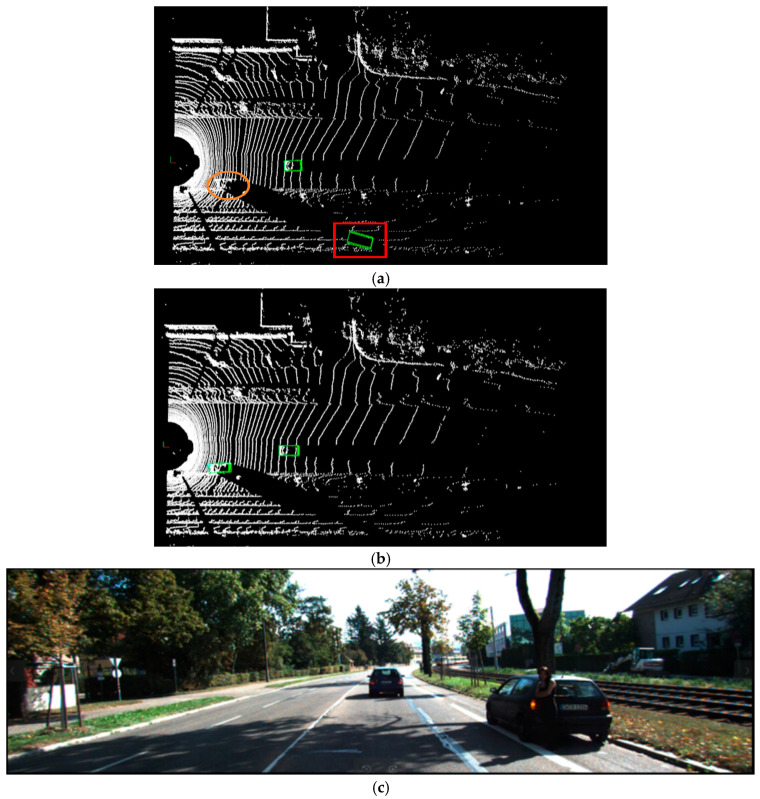
Visualization of LiDAR point cloud detection results under the easy difficulty level: (**a**) Results from PointPillars; (**b**) Results from the proposed improved algorithm; (**c**) RGB images from real-world scenes.

**Figure 6:**
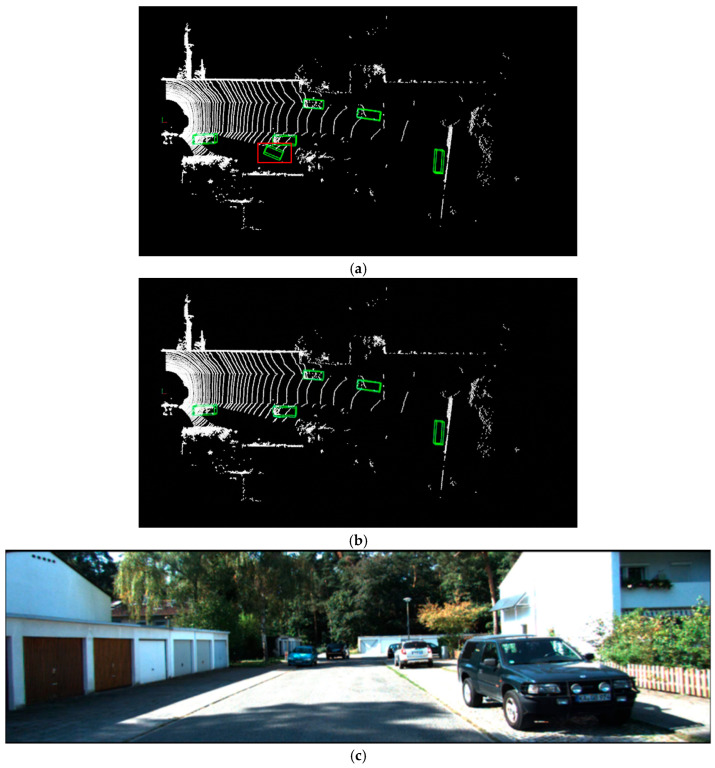
Visualization of LiDAR point cloud detection results under the moderate difficulty level: (**a**) Results from PointPillars; (**b**) Results from the proposed improved algorithm; (**c**) RGB images from real-world scenes.

**Figure 7:**
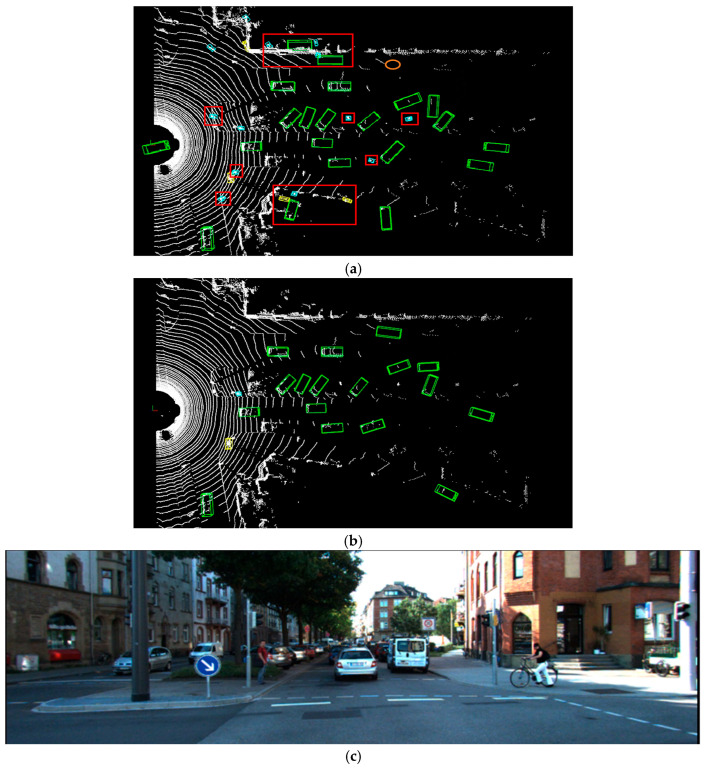
Visualization of LiDAR point cloud detection results under the hard difficulty level: (**a**) Results from PointPillars; (**b**) Results from the proposed improved algorithm; (**c**) RGB images from real-world scenes.

As illustrated in Figure 5, Figure 6 and Figure 7, this paper compares the detection performance of the proposed algorithm and the baseline PointPillars algorithm under easy, moderate, and hard scenarios on the KITTI dataset. The upper half of each figure (Figure 5a,b, Figure 6a,b and Figure 7a,b) presents the 3D object detection results in the point cloud view, while the lower half (Figure 5c, Figure 6c and Figure 7c) displays the corresponding RGB camera images from real-world scenes. In the 3D detection results, cars, pedestrians, and cyclists are represented by green, blue, and yellow bounding boxes, respectively. Red rectangular borders indicate false positives (i.e., incorrectly detected objects), whereas orange borders indicate false negatives (i.e., missed detections).

Figure 5 reveals missed detections of cars and pedestrians by the PointPillars algorithm in easy scenarios. The orange oval border highlights missed detections of nearby car and pedestrian under low-light conditions. The red rectangular border indicates misclassifications, where distant non-car object is erroneously identified as car. As shown in Figure 6, in moderately complex scenes, red rectangular border denotes misclassified cars, where square fences are mistakenly detected as a car due to object occlusion. Notably, the proposed algorithm effectively avoids both misclassifications and missed detections in these scenarios.

**Figure 8:**
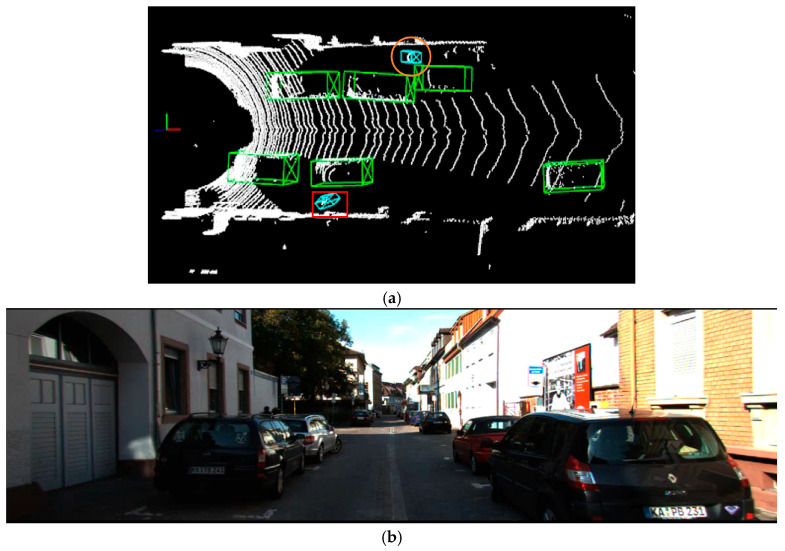
(**a**) Detection results under extreme occlusion condition; (**b**) RGB images from real-world scenes.

As shown in Figure 7, the red rectangular borders highlight a high false positive rate of the PointPillars algorithm when detecting objects in hard scenes. Roadside traffic signs, streetlights, and other structures are erroneously classified as pedestrians, while bicycles parked along the roadside are misidentified as cyclists. Additionally, distant cars are sometimes falsely detected, with square walls mistakenly recognized as cars. Some partially occluded cars are also missed. The proposed algorithm effectively mitigates the false positives and missed detections for small objects in hard scenarios, yielding improved visualization results. As shown in Figure 8, false detections and missed detections of small objects occur under extreme occlusion conditions.

### 4.4. Ablation Study

To further assess the impact of the proposed improved modules on the performance of the 3D object detection network, a series of ablation studies are conducted. These studies evaluate the effects of data-level fusion, the SAM, and the PCA module on detection accuracy. The evaluation metrics include the average precision (AP) for three categories—Car, Cyclist, and Pedestrian—as well as the mean Average Precision (mAP) averaged across these categories. Table 5 and Table 6, respectively, present the BEV and 3D detection performance comparisons for three object categories—Car, Pedestrian, and Cyclist—in the ablation study.

The baseline is the original PointPillars algorithm. In Experiment 1, semantic information extracted from images is fused into the original point clouds, yielding an improvement in mAP. Experiment 2 introduced the SAM into the backbone network, resulting in enhanced detection accuracy for small objects. Experiment 3 introduced the PCA module into the PFN layer, resulting in decreased detection accuracy for small objects.

In Experiment 4, the SAM is added to the PointPillars Backbone network that incorporates image semantic information. Pedestrian detection accuracy increases by 5.85% and 4.49% in the BEV and 3D perspectives, respectively. However, cyclist detection accuracy experiences a slight decrease. This reduction is attributed to the ambiguous semantic definition of cyclists, who typically consist of both “riders” and “bicycles.” Semantic segmentation for cyclists often suffers from fragmentation, blurred boundaries, or misclassification in images. Consequently, the projected semantic features on the point clouds appear spatially discontinuous or fragmented. This spatial inconsistency challenges the SAM’s ability to accurately identify the cyclist region as a salient area during BEV feature weighting, thereby slightly reducing detection accuracy.

**Figure 9:**
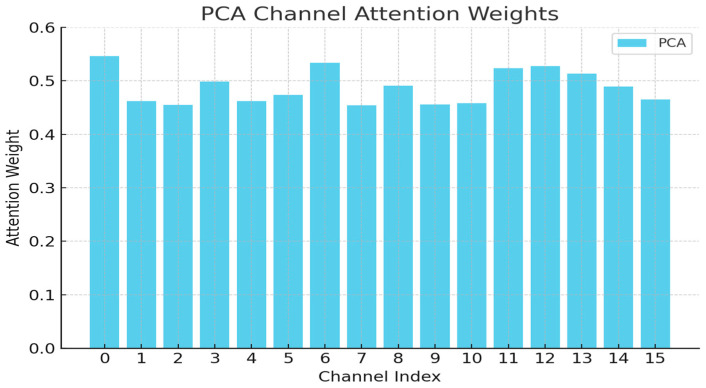
PCA channel attention weights.

Compared to Experiment 3, Experiment 5 incorporates the PCA module after integrating semantic information, leading to improvements in both pedestrian and cyclist detection accuracy. However, directly introducing the PCA module into the original PointPillars network results in a decline in small-object detection performance. As shown in Figure 9, the PCA weight visualization indicates a balanced distribution across channels, without significantly suppressing any specific channel. In Experiment 3, the decline in small-object performance is primarily attributed to the inherently weak features of small objects in the original point cloud, with PCA’s equalization further reducing the relative importance of these channels. The balancing effect of the PCA module reduces the relative prominence of the small-object channels. These observations indicate that the PCA module is better suited for multi-modal features enhanced with semantic information.

To further enhance the network’s performance in detecting small objects with complex semantic structures, particularly cyclists, this paper proposes a 3D object detection algorithm that leverages a dual-attention mechanism combined with multimodal data fusion. The algorithm integrates image semantic information with raw point clouds and incorporates the PCA module and the SAM within the downsampling stages of the PFN layer and backbone network, respectively. Under the BEV evaluation metric, the AP for pedestrians and cyclists increases by 7.06% and 3.08%, respectively, leading to a 3.32% improvement in mAP. Under the 3D evaluation metric, the AP values increase by 4.36% and 2.58%, with the mAP improving by 2.25%.

The inference speed of the model is summarized in Table 7. Experimental results demonstrate that the introduction of the SAM and the PCA module incurs only marginal computational overhead and latency, indicating that both modules are lightweight and suitable for real-time deployment. Although the incorporation of image semantic fusion introduces additional costs, the proposed method still achieves 50 FPS, which is sufficient to meet the real-time requirements of autonomous driving. In future work, the method will be deployed on other hardware platforms for further evaluation.

## 5. Conclusions and Future Work

### 5.1. Conclusions

This paper proposes an improved 3D object detection algorithm based on dual-attention mechanism for multimodal data fusion, aimed at addressing the challenge of poor detection performance for small objects in 3D detection tasks. First, the original point clouds are fused with image semantic information to generate a painted point cloud, thereby enriching the semantic content of the point clouds and reducing false detections of small objects. Second, to overcome the insufficient feature representation of small object regions during the conversion of point clouds into pseudo-image representations via the PFN layer, the PCA module is designed. This module enables the network to adaptively learn and dynamically adjust channel-wise feature weights, emphasizing key channels to enhance feature representation for small objects and reduce detection failures. Finally, the SAM is incorporated into the backbone network to highlight salient regions in the BEV feature map, further improving feature extraction for small object detection.

In addition to accuracy improvements, the proposed method maintains a high level of computational efficiency. By employing lightweight attention modules and optimizing channel-wise operations, the framework achieves enhanced detection performance without significantly increasing inference time or resource consumption. Experimental evaluation on the KITTI dataset validates the effectiveness of the proposed approach, with notable improvements in both BEV and 3D object detection tasks. Specifically, pedestrian and cyclist detection achieved significant gains across all difficulty levels, underscoring the robustness and practical potential of the method in real-world autonomous driving scenarios.

Although the proposed method has achieved notable improvements, it still exhibits certain limitations under extreme occlusion and adverse weather conditions such as rain and snow. In these challenging scenarios, object features become less distinguishable, leading to a decline in detection accuracy.

### 5.2. Future Work

Enhancing Model Robustness in Complex Scenarios: In future work, we will extend the evaluation of the proposed method to a wider range of challenging real-world conditions, including adverse weather and diverse lighting environments. Furthermore, we plan to investigate advanced multimodal fusion strategies, adaptive attention mechanisms, and refined preprocessing techniques to further enhance the detection of small and heavily occluded objects.

Adaptive Modality Selection and Weighting: In real-world autonomous driving scenarios, the relative reliability of different sensor modalities can vary substantially under changing environmental conditions. For example, visual information may deteriorate in low-light or adverse weather, whereas LiDAR data can become sparse or noisy during heavy rain or snowfall. To mitigate these issues, future research could investigate adaptive modality selection and dynamic weight allocation strategies, allowing detection frameworks to automatically adjust the contribution of each modality in response to contextual scene conditions.

## Figures and Tables

**Table 1:** KITTI dataset categorization.

Difficulty Level	Object Size	Truncation Level	Occlusion Level
Easy	Large (≥40 px)	Low (≤15%)	None or Partial
Moderate	Medium (≥25 px)	Moderate (≤30%)	Partial
Hard	Small (≥25 px)	High (≤50%)	Heavy

**Table 2:** Comparison of BEV metrics on the KITTI dataset (%).

	Car			Cyclist			Pedestrian		
	Easy	Mod	Hard	Easy	Mod	Hard	Easy	Mod	Hard
SECOND	88.07	79.37	77.95	73.67	56.04	48.78	55.10	46.27	44.76
F-PointNet	88.70	84.00	75.33	75.38	61.96	54.68	58.09	50.22	47.20
MVX-Net	89.20	85.90	78.10	N/A	N/A	N/A	N/A	N/A	N/A
PointPillars	92.24	87.94	85.22	84.69	66.21	61.82	54.62	48.81	44.84
PillarNet	89.70	86.96	84.60	83.42	67.01	62.86	58.91	53.48	50.90
VoxelNextFusion	94.97	91.31	89.06	83.00	69.93	63.71	61.71	51.30	47.89
Ours	91.50	87.93	85.36	86.49	69.98	65.44	62.54	55.55	51.35

**Table 3:** Comparison of 3D metrics on the KITTI dataset (%).

	Car			Cyclist			Pedestrian		
	Easy	Mod	Hard	Easy	Mod	Hard	Easy	Mod	Hard
SECOND	83.13	73.66	66.20	70.51	53.85	46.90	51.07	42.56	37.29
F-PointNet	81.20	70.39	62.19	71.96	56.77	50.39	51.21	44.89	40.23
MVX-Net	83.20	72.20	65.20	N/A	N/A	N/A	N/A	N/A	N/A
PointPillars	86.96	76.01	72.78	79.98	60.20	55.74	49.26	43.36	39.32
PillarNet	87.69	77.81	74.60	80.75	62.50	58.83	51.58	46.80	44.39
SMS-Net	87.01	76.21	70.45	75.35	60.23	53.37	53.46	44.76	41.35
VoxelNextFusion	90.40	82.03	79.86	79.28	64.47	58.25	52.56	45.72	41.85
Ours	86.19	75.90	73.04	81.92	63.05	58.66	54.52	47.67	42.84

**Table 4:** Performance comparison of PCA and SE attention modules.

Module	Parameters	FLOPs	BEV (mAP)%	3D (mAP)%
PCA	1024	32.8 M	64.87	57.39
SE	512	27.4 M	62.99	54.61

**Table 5:** Impact of different modules on BEV detection performance (%).

	Fusion	SAM	PCA	Car	Pedestrian	Cyclist	mAP
Baseline				88.47	49.42	70.90	69.59
Experiment 1	√			88.69	53.99	67.63	70.10
Experiment 2		√		88.47	53.12	71.31	70.97
Experiment 3			√	88.23	51.05	68.22	69.17
Experiment 4	√	√		88.04	55.26	70.94	71.41
Experiment 5	√		√	88.98	55.77	73.97	72.90
Our method	√	√	√	88.27	56.48	73.98	72.91

**Table 6:** Impact of different modules on 3D detection performance (%).

	Fusion	SAM	PCA	Car	Pedestrian	Cyclist	mAP
Baseline				78.59	43.98	65.30	62.62
Experiment 1	√			78.60	46.27	65.11	63.32
Experiment 2		√		77.67	46.65	67.91	64.07
Experiment 3			√	78.44	42.80	65.59	62.27
Experiment 4	√	√		78.25	48.47	64.50	63.74
Experiment 5	√		√	78.96	46.91	65.31	63.73
Our method	√	√	√	78.38	48.34	67.88	64.87

**Table 7:** Inference speed of the model.

	Fusion	SAM	PCA	FPS	Speed/(s·frame^−1^)
Baseline				115	0.0087
Experiment 1	√			48	0.0210
Experiment 2		√		114	0.0088
Experiment 3			√	110	0.0091
Experiment 4	√	√		49	0.0206
Experiment 5	√		√	49	0.0202
Our method	√	√	√	50	0.0199

## Data Availability

The datasets used and analyzed in this study are publicly available in the KITTI Vision Benchmark Suite: http://www.cvlibs.net/datasets/kitti/ (accessed on 10 November 2024). No new datasets were generated during the current study.
